# Barriers and facilitators to health technology adoption by older adults with chronic diseases: an integrative systematic review

**DOI:** 10.1186/s12889-024-18036-5

**Published:** 2024-02-16

**Authors:** Alessia Bertolazzi, Valeria Quaglia, Ramona Bongelli

**Affiliations:** Department of Political Science, Communication and International Relations, University of Macerata, Don Minzoni street, 22, 62100 Macerata, Italy

**Keywords:** Older adults, Technology adoption, Chronic disease, Health technology, Telemedicine, Health information technology

## Abstract

**Background:**

In recent years, healthcare systems have progressively adopted several technologies enhancing access to healthcare for older adults and support the delivery of efficient and effective care for this specific population. These technologies include both *assistive technologies* designed to maintain or improve the independence, social participation and functionality of older people at home, as well as *health information technology* developed to manage long-term conditions. Examples of such technologies include telehealth, wearable devices and mobile health. However, despite the great promise that health technology holds for promoting independent living among older people, its actual implementation remains challenging.

**Methods:**

This study aimed to conduct an integrative systematic review of the research evidence on the factors that facilitate or hinder the adoption of different types of technology by older individuals with chronic diseases. For this purpose, four electronic databases (PsycArticles, Scopus, Web of Science and PubMed) were queried to search for indexed published studies. The methodological quality of the selected papers has been assessed using the Mixed Methods Appraisal Tool (MMAT).

**Results:**

Twenty-nine articles were selected, including 6.213 adults aged 60 or older. The studies have been synthesised considering the types of technological interventions and chronic diseases, as well as the main barriers and facilitators in technology acceptance. The results revealed that the majority of the selected articles focused on comorbid conditions and the utilisation of telemedicine tools. With regard to hindering and facilitating factors, five main domains were identified: demographic and socioeconomic, health-related, dispositional, technology-related and social factors.

**Conclusion:**

The study results have practical implications not only for technology developers but also for all the social actors involved in the design and implementation of healthcare technologies, including formal and informal caregivers and policy stakeholders. These actors could use this work to enhance their understanding of the utilisation of technology by the ageing population. This review emphasises the factors that facilitate technology adoption and identifies barriers that impede it, with the ultimate goal of promoting health and independent living.

**Supplementary Information:**

The online version contains supplementary material available at 10.1186/s12889-024-18036-5.

## Background

Over the last few decades, the elderly population has grown significantly, and it is projected that the proportion of people aged 65 and above will continue to increase from 10% in 2022 to 16% in 2050 [1]. This demographic shift has led to increased pressure on healthcare systems’ ability to plan and provide effective healthcare services for older adults. In fact, the ageing of the population has resulted in an increase in long-term diseases such as diabetes, chronic respiratory diseases (e.g., chronic obstructive pulmonary disease and asthma), neurological disorders (e.g., dementia, Alzheimer’s disease and Parkinson’s disease) and cardiovascular disease (e.g., ischaemic heart disease, cerebrovascular disease and hypertensive heart disease) [2]. Along Europe, 72.5% of people aged 85 years or older reported the presence of at least one health problem [3]. In the U.S., 23.9% of the population aged 65 or older has one chronic condition, while 63.7% has two or more [4]. The growing prevalence of multimorbidity is associated with increased utilisation and cost of healthcare services [5]. The growth of the elderly population with multiple long-term diseases has implications not only at the societal level but also at the individual level. Older people have specific health needs that need to be met in a timely manner, as complications and limitations related to illness can impact their independence, autonomy and overall well-being [6].

However, this social group faces specific difficulties in accessing healthcare services. Several studies have investigated the factors influencing access to healthcare. These studies suggest that sociodemographic determinants (e.g., female gender, older age, etc.) [7], age-related factors (e.g., limited mobility, sensory impairments, and disability) [8], socioeconomic variables (such as lower income, lack of complementary insurance, cost, and transportation) [7, 9], as well as organisational features of healthcare systems (e.g., extended waiting periods for medical examinations) [10], play significant roles in influencing access to healthcare.

To overcome these barriers, healthcare systems have progressively implemented various types of *digital health technologies* aimed at enhancing elderly care. The literature presents various terminologies in this regard. The World Health Organization defined digital health as ‘the field of knowledge and practice associated with the development and use of digital technologies to improve health’ [11, 12]. Particularly, digital technology refers to both the software, which includes computer coding programmes that provide instructions for computer operations, and the hardware, which consists of physical computer devices. These components work together using digital coding, also known as binary coding. Additionally, digital technology encompasses the infrastructure that supports these software and hardware components [13].

Furthermore, specific terms have been coined to describe digital health technologies for older adults. For instance, the umbrella term ‘gerontechnology’ has emerged to define the set of technologies intended to promote the independence of older adults, facilitate ageing in place and accommodate age-related declines and impairments [14]. These technologies include both assistive technologies designed to maintain or improve the independence, social participation and functionality of older people at home, as well as health information technology for managing long-term conditions. Examples of these technologies include telehealth, wearable devices, and mobile health [15, 16]. In the Nordic European welfare state systems, the term ‘welfare technology’ has been coined to emphasise the public and universalistic nature that these devices should have. This term refers to a range of digital tools with integrated platforms that are adopted by public care services to promote welfare among individuals [17].

In this paper, the expression ‘health technologies’ will be employed to encompass a range of digital technologies designed for the management of health conditions. These include the electronic health record, mobile health apps, wearable devices, telehealth and telemedicine [11, 12].

Health technologies can be applied in multiple areas, such as self-management of chronic diseases and the sharing and transfer of clinical data. These applications can improve adherence to therapeutic regimens, facilitate communication with healthcare professionals and enable timely interventions. Previous research on telemedicine has shown that it can reduce travel time and costs, making it an important resource for older patients living in underserved areas. It can also diminish patients’ waiting times for medical encounters, thus shortening the time for diagnosis. Lastly, especially during pandemic times, it can also reduce the risk of contagion and infections [18, 19]. Moreover, health technologies can promote aging in place, enabling older people to safely remain in their homes (or in appropriate housing, depending on their health conditions), thereby reducing hospitalization and avoiding institutionalization. A recent systematic review revealed that smart home technologies used for the management of chronic diseases in older adults can improve several health outcomes [20]. The monitoring of daily living activities, such as mobility, posture, falls or sleeping disorders can facilitate personalized and timely interventions. Furthermore, it promotes physical activity, enhances the quality of life and fosters a sense of security and well-being in older people. Other instruments, such as external memory aids and telemedicine, can support chronic patients in managing medication, or enhance the control of vital signs [20].

However, despite the great promise of health technology in enabling older people to maintain their independence for longer, its actual implementation remains challenging. The literature highlights several factors that may have a negative impact on the adoption of health technologies in the elderly population.

One line of research has focused on socio-structural characteristics that may increase inequalities in technology use. Previous studies have found that older individuals with lower income and education levels have limited access to broadband, lower health literacy and lower digital competencies, which in turn leads to limited technology adoption [21–24].

The second research line has concentrated on individual factors that may influence the acceptance of technology, such as age or health conditions. Cognitive deficits, as well as physical impairments (e.g., vision and hearing loss and mobility limitations), can pose significant challenges in the use of technology [15, 25]. In addition, other studies have examined the attitudes of the elderly towards health technologies. For instance, some of them have focused on the strategies employed by individuals to resist stereotypes associated with old age. The rejection of technology may indeed be associated with negative (self)perceptions related to the loss of dignity and autonomy, as well as the fear of being stigmatised as someone no longer able to take care of oneself [26–28]. Furthermore, perceptions of the usefulness and ease of use of technology, as well as beliefs about privacy, may influence its adoption. While privacy concerns have not been observed for some technologies, such as telemedicine [26], they could be a barrier for other devices, including fall detection or bed occupancy sensors [29, 30].

The third line of study focused on aspects related to the technology itself, including the role of users in the technology design process and the socio-material characteristics of the technology, as well as users’ adaptation strategies. Regarding the first aspect, while participatory technology design methods are becoming more widespread, there are difficulties in implementing these methods in practice. Users are often still perceived as passive consumers, and their needs may not be fully addressed [31, 32]. Concerning the second aspect, research has emphasised that in order to understand the strategies for the adoption or refusal of technologies, they should be considered within the context of the usual practices of the elderly population [33]. In fact, research has shown that older people tend to adapt technologies to suit their needs through ‘bricolage’ arrangements, using devices in ways that were not originally intended [33–35].

Across these strands of research, however, tension emerges between two different perspectives regarding the impact that health technologies would have on social actors. On one side, there is the self-surveillance effect of these technologies, while on the other side, there is the empowerment effect that would be embedded in health technologies [36–38]. The first perspective emphasises the disciplinary effects of health technologies, which could engender behavioural changes through continuous data generation and transmission [39–42]. This ultimately would promote both an expansion of the ‘medical gaze’ into the everyday lives of self-tracked patients [37] and an individualistic dimension of health, shifting the responsibility from healthcare systems to individuals [43, 44].

The second point of view presents the individualisation of responsibility for one’s health condition in a positive manner and highlights the empowering potential of technologies. Health technologies empower patients by instilling in them a greater awareness of their health status. This awareness would thus lead to a greater sense of responsibility for one’s own health and trigger a virtuous cycle [45–46].

Therefore, although much research has been conducted, a variety of factors is at play in the acceptance of technology by older adults. Additionally, there are ambivalent points of view about the impact of health technologies on people. Upon examining recent reviews, it appears that studies are narrow in focus, as they only consider one set of factors at a time or a single technology, or they are not systematic and are based on a scoping review [47]. Particularly, the research appears to be limited to specific technologies, such as falls-prevention interventions [48], m-Health technology [49], telemedicine [18, 50] and electronic personal health records [51]. Moreover, it solely examines hurdles and barriers without considering facilitating factors [51]. It also fails to indicate the specific medical condition or set of conditions that the technology is targeted at [47, 52, 53].

To shed light on this topic, the present integrative systematic review aims to identify barriers and facilitators that impact the adoption of different health technologies by older individuals with chronic diseases. Considering the multitude of diseases that could be included in the review and the wide range of technologies addressed in the health management of elderly individuals with chronic conditions, we have chosen to adopt a broad conceptualisation of ‘facilitators’ and ‘barriers’. With the first term, we refer to factors that support the adoption of technology and provide an incentive to continue using it. Additionally, we consider factors that have been identified in the literature as not hindering the utilisation of technology. Barriers, on the other hand, consist of all the elements that hinder the adoption of technology or discourage its use.

Thus, the research questions that guided the review were as follows:

### RQ1

What are the main factors hindering the adoption of technology by older adults with chronic diseases?

### RQ2

What are the main factors facilitating the adoption of technology by older adults with chronic diseases?

## Method

An integrative systematic review was conducted by implementing a search strategy that allowed for a comprehensive examination of the barriers and facilitators to the adoption of chronic disease-related technology in the elderly population. This method can have direct applicability for practical implementation and policymaking, and ‘allows for the inclusion of diverse methodologies (e.g., experimental and non-experimental research)’ [54]. Therefore, our analysis includes qualitative research, randomised and non-randomised quantitative studies and mixed method studies. It also comprises papers focused on different technologies and different types of chronic diseases.

### Inclusion and exclusion criteria

In order to identify eligible articles, inclusion and exclusion criteria were established before starting the literature search. These criteria were based on the exploratory research questions in the review. The pre-defined inclusion criteria for study selection were as follows: (a) the sample must include participants aged 60 or older; (b) the sample must include participants affected by chronic disease; (c) the studies must focus on facilitators or barriers to the adoption of technologies related to chronic disease management; (d) the studies must be empirical and use qualitative, quantitative or mixed methods; (e) the studies must be published in English; (f) the studies must focus on technology targeting older people. The exclusion criteria adopted were as follows: (a) mixed sample population with participants above and below 60 years of age; (b) publications such as theoretical contributions, letters to the editor, systematic or scoping reviews, dissertations, conference proceedings, or those adopting non-standardised techniques and lacking sufficient analytical rigour (e.g., narrative reviews); (c) studies that evaluated a healthcare service instead of digital technology tools enabling the service (e.g., studies that evaluated the general telemedicine service without focusing on the platform enabling telemedicine services).

### Search strategy

Four electronic databases were queried to search for published studies: PsycArticles, Scopus, Web of Science and PubMed. Records published between January 2012 and April 2022 were considered, operating with the following PICo framework [55] and using a Boolean search strategy through keywords and Medical Subject Headings (MeSH) terms (see Table 1). The full search strategy can be found in Additional file 1.

**Table 1:** Identification of search terms using the PICo mnemonic applied to the research question

Population	Phenomena of interest	Context
Elderly OR older adult OR ageing people	Technology OR gerontechnology	Chronic disease OR chronic illness OR long-term conditions OR chronic conditions

The timeframe was chosen based on the findings from previous reviews [47; 53] and the evolution of the digital health tools under consideration. Publications reporting was guided by the Preferred Reporting Items for Systematic Reviews and Meta-Analyses Statement (PRISMA) flow diagram [56].

**Fig. 1:**
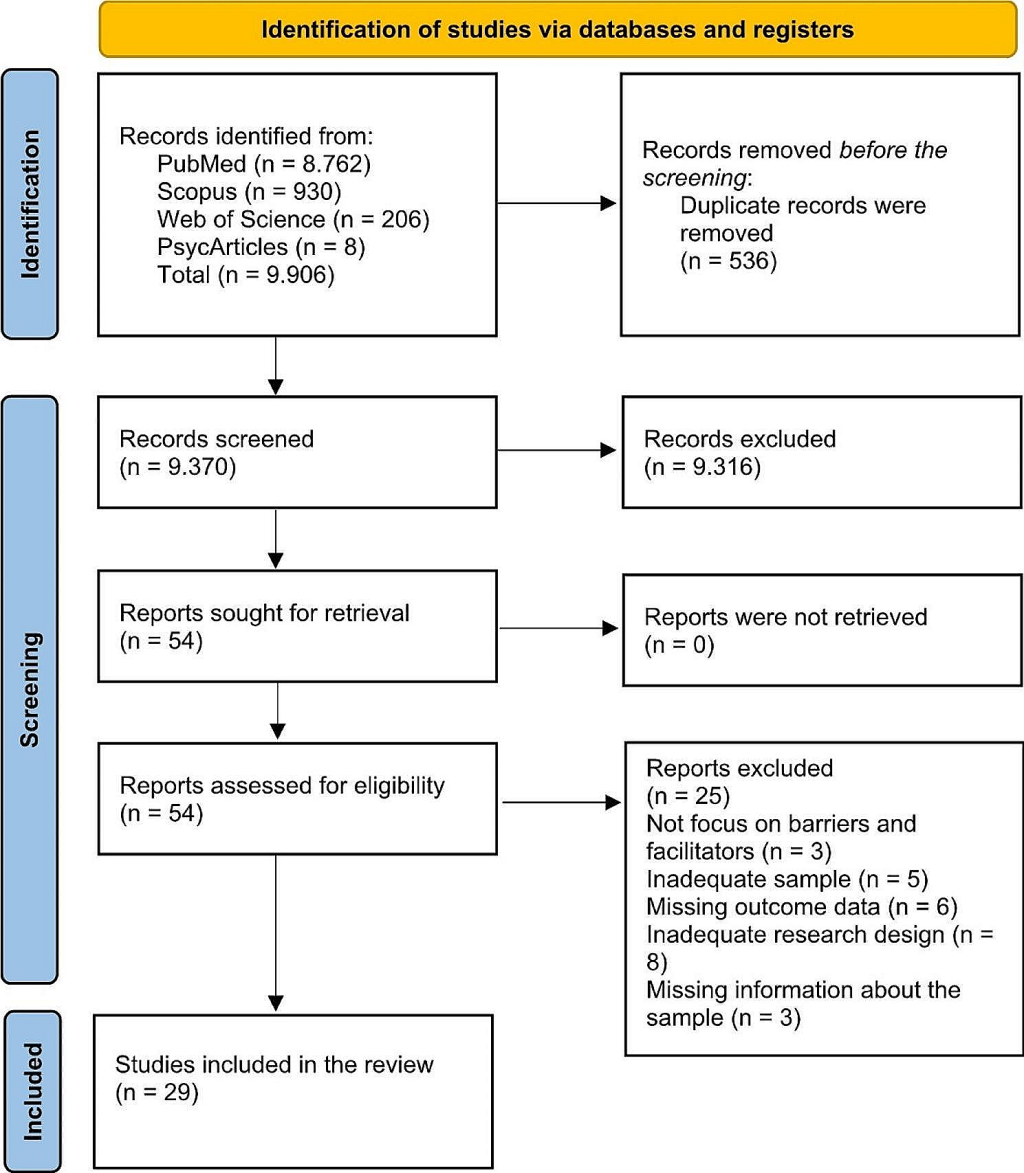
PRISMA flow diagram

As shown in Fig. 1, a total of 9.906 publications have been retrieved from the databases. After removing the duplicates, 9.370 have been screened. All the authors reviewed the titles and abstracts of the records to identify relevant studies that met the inclusion/exclusion criteria. Disagreements were resolved through discussions until a consensus was reached.

### Data extraction and analysis

As a result of the first screening, fifty-four publications were identified for possible inclusion. The methodological quality of the selected records has been assessed using the Mixed Methods Appraisal Tool (MMAT) [57]. All authors independently undertook the quality assessment process by performing blind MMAT evaluations of the same articles and comparing the results.

Regarding data extraction, the full texts of the included articles were obtained to extract pertinent details such as the authors’ names, year of publication, study objectives, study design, country of study, study setting (e.g., home or hospital), type of chronic disease and type of technology used. Each author independently identified facilitators and barriers through the content analysis of the included articles. One author independently (A.B.) grouped together homogeneous factors and redefined similar factors with different names. The final list of factors was discussed among authors until reaching a consensus. To synthesise and organise the results more effectively, these factors were further grouped into macro-categories, which are referred to as domains. The five identified domains emerged through a combination of inductive and deductive approaches. Some domains were deduced from previous systematic reviews [47, 51], including technology-related factors and social factors. Some factors emerged inductively from the data itself, such as socioeconomic factors, health-related factors and dispositional factors.

## Results

During the quality assessment process, the authors agreed to exclude twenty-five articles for various reasons. These reasons included poor methodological quality, inadequate focus on barriers and facilitating factors (such as factors that were mere inferences of the authors) or articles that solely presented protocols for developing technology. Twenty-nine articles were selected, including 6.213 adults aged 60 or older. The characteristics of the screened studies are shown in Additional File 2. As for the nomenclature of technological interventions, the original designations used within each article have been preserved to avoid inappropriate simplifications that could have resulted from their reclassification. Regarding the research design of the included studies, fifteen were based on research trials, presenting different lengths of the evaluation period: two weeks [58], five weeks [59], two months [60–62], three months [63, 64], nine months [65], one year [66, 67], and fifteen months [68]. However, one trial was conducted in a laboratory [69], so no evaluation period was planned. Additionally, three studies relied on data derived from prior trials, with subsequent secondary analyses performed [70–72]. The remaining articles had adopted different methodologies, encompassing qualitative approaches– such as interviews [73–76], focus groups [77–79], or both [80]–, quantitative ones through cross-sectional studies [81–84], or mixed methods [85].

The analysis of the included articles focused on (a) types of technological interventions, (b) types of chronic diseases and (c) the main barriers and facilitators in technology acceptance.

Concerning the types of technological interventions for managing chronic diseases, eight studies have examined telemedicine, which includes telecare, telemonitoring, telehealth, and telerehabilitation programmes [60–63, 65, 67, 71, 86]. Seven studies have focused on digital health platforms, such as web portals and video conferencing for home-based education [59, 64, 66, 78, 79, 83, 85]. Five studies have explored wearable technology, including the use of pedometers and self-tracking technology [58, 72, 73, 75, 77]; instead, m-health services have been examined by three studies [70, 74, 84]. Three studies have investigated information and communication technology (ICT) [80–82], that is services obtained through ICT (e.g., messaging services, using email to communicate with doctors, medication services and reminders, online tools, etc.); instead, one article focused on the Internet for health information seeking [71], as it specifically refers to the Internet exclusively for searching for health information online. Lastly, two articles have examined home assistive technologies (smart home) [68, 69], two studies have focused on assistive robots [69, 76], and one on active video games [75].

In terms of the specific chronic diseases targeted by technological interventions, 16 articles included aged people with multiple chronic conditions for technological intervention [59–61, 63, 65–67, 69, 73, 78–80, 82, 83, 85, 86]. While, other articles focused on a specific chronic disease: COPD [71, 72, 75], cognitive impairments [68, 70, 76], heart failure [74, 84], Parkinson’s disease [77, 81], hypertension [58], vestibular dysfunction [64], and diabetes [62].

The identified barriers and facilitators are shown in Table 2.

**Table 2:** Facilitators and barriers to technology adoption

Facilitators	Barriers
**Demographic and socioeconomic factors**	
*Age* - Mobile application [84] - Pedometer [72] - Telemedicine [62]	*Older age* - ICT [64; 66]
*Higher level of education* - ICT [82]	*Low education* - Telehealth [71] - Internet for online health information [71]
	*Limited/fixed income* - mHealth [74]
*Cost-effectiveness* - Multi-site videoconferencing for home-based education [59] - mHealth [74] - Smart home technology and a socially assistive robot [69] - Home assistive technology services [65] - Patient portal [79]	*Cost* - mHealth [74] - Blood pressure monitor [58] - Computer-Based Self-Management System [85]
	*Limited space at home for the system* - Computer-Based Self-Management System [85]
**Health-related factors**	
	*Poor learning skills* - Telehealth [71] - Internet for online health information [71]
*Cognitive ability* - ICT [82]	*Cognitive impairments [e.g., poor memory and cognitive dissonance]* - Telehealth [71] - Internet for online health information [71] - Blood pressure monitor [58] - Robots and sensors [76]
	*Sensory deficits [e.g., poor vision and hearing impairments]* - mHealth [74] - ICT [82] - Internet for online health information [71] - Robots and sensors [76]
*Awareness and a better understanding of the illness* - Telehealth [71] - Telemonitoring [65] - Telecare [67]	*Severe anxiety about the illness* - Telemonitoring [65]
*Adoption of technology in the early stages of the disease* - Home assistive technology services [65]	*Complexity of health conditions* *[e.g., comorbidities and worsening of the condition]* - Telerehabilitation [60] - Digital health platform [66]
	*Type of medical condition* *[e.g., depressive and anxiety symptoms, diabetes and heart disease]* - Telemonitoring [65] - Telemedicine [86]
*Be monitored continuously by healthcare providers and receive timely care* - Wearable technology for Parkinson’s disease [77] - Digital health platform [66] - Telemonitoring [65] - Wearable activity trackers [75]	
*Improving the self-management of chronic diseases* - Digital health platform [66] - Telerehabilitation [60] - Computer-Based Self-Management System [85]	
*Perceive an improvement in medical condition, lifestyle and quality of life* - Digital health platform [66] - Smart home technology and a socially assistive robot [69] - Computer-Based Self-Management System [85]	
**Dispositional factors**	
*Trustworthiness and reliable information* - Smart home technology and a socially assistive robot [69]	*Scepticism about the accuracy of the results* - Health information technologies for self-tracking [73] - Digital health platform [66] - Telehealth [71]
	*Judging oneself or being judged negatively by physicians when one does not monitor data* - Health information technologies for self-tracking [73]
*Willingness to learn* - mHealth [74]	*Aversion to/difficulty learning how to operate new technology* - Telerehabilitation [60] - ICT [80] - Home assistive technology services [65] - Patient portal [79] - Active video games [75]
	*Fear of using new technology* *[e.g., fear of making mistakes, malfunctioning and online fraud]* - Internet for online health information [71] - Telehealth [71] - ICT [80] - Blood pressure monitor [58] - Active video games [75]
*Technological self-efficacy* - ICT [82] - Patient portal [79]	*Lack of confidence and technical skills/unfamiliarity with the technology/computer anxiety* - mHealth [70, 74] - Digital health platform [66] - Internet for online health information [71] - Telehealth [71] - Computer-Based Self-Management System [85] - Patient portal [79] - Portal technology for self-care [83]
*Previous experience with the technology* - mHealth [74] - ICT [80] - Portal technology for self-care [83]	*Previous negative experiences with the technology* - Patient portal [79]
	*Lack of need for technology* - mHealth [74]
*Motivation/interest to use the technology* - ICT [80] - Portal technology for self-care [83]	*Lack of motivation, interest or personal inertia* - Telerehabilitation [60] - Internet for online health information [71] - Telehealth [71] - Patient portal [79] - Computer-Assisted Home Training Programme [64] - Robots and sensors [76]
	*Established routines and a conservative mentality* - Telehealth [71] - Patient portal [79] - Robots and sensors [76]
*Personal enjoyment* - Active video games [75]	*Competitive characteristics* - Wearable activity trackers/Active video games [75]
*Perceived usefulness* - mHealth [74] - Blood pressure monitor [58] - Computer-Based Self-Management System [85] - Portal technology for self-care [83] - Active video games [75]	*Perception of technology as demanding, time-consuming and not useful* - Health information technologies for self-tracking [73] - Digital health platform [66] - Active video games [75]
*Higher level of health literacy* - Portal technology for self-care [83]	
*Technical factors*	
*Perceived ease of use* - Multi-site videoconferencing for home-based education [59] - mHealth [74] - ICT [82] - Internet for online health information [71] - ICT [80] - Blood pressure monitor [58] - Computer-Based Self-Management System [85] - Patient portal [79] - Portal technology for self-care [83] - Telemedicine [61, 62]	*Technical issues* *[e.g., connectivity, audio problems, low-quality graphics, difficult navigation, inappropriate alerts and logging in]* - Multi-site videoconferencing for home-based education [59] - Telerehabilitation [60] - Telemonitoring [65] - Computer-Based Self-Management System [85] - Patient portal [79] - Robot [76]
*Privacy is not a concern* - Wearable technology for Parkinson’s disease [77]	*Privacy and safety concerns* - Internet for online health information [71] - Patient Web Portal [78] - Portal technology for self-care [83]
*Comfortability of wearable technology* - Wearable technology for Parkinson’s disease [77]	
*Patient involvement in the design process* - Blood pressure monitor [58]	*Scarce involvement of patients in technologies development* - Wearable technology for Parkinson’s disease [77]
*Self-tracking functions* *[e.g., to set measurable goals, to receive reminders and to quantify activities]* - Health information technologies for self-tracking [73] - Telerehabilitation [60] - Patient Web Portal [78] - Telecare [67] - Wearable activity trackers [75]	*Medical data reminded patients of the negative aspects of their illness* - Health information technologies for self-tracking [73]
*Adequate training* - mHealth [74] - Telehealth [63, 71] - Blood pressure monitor [58]	*Lack of training or instruction on the use of the technology* - Wearable activity trackers/Active video games [75]
*Likeable appearance* - Smart home technology and a socially assistive robot [69] - Robot [76]	*Poorly designed interface* - mHealth [74] - Computer-Based Self-Management System [85]
*Clarity in the presentation and organisation of information* - Blood pressure monitor [58] - Computer-Based Self-Management System [85]	*Overload/complicated information* - Patient Web Portal [78]
	*Not having the required equipment [e.g., a game console]* - Active video games [75]
*Natural speech and eye contact with the robot* - Smart home technology and a socially assistive robot [69]	*A lack of interactive design features* - Telemonitoring [65]
*Technical helpdesk* - Digital health platform [66]	*Lack of technical support* - Portal technology for self-care [83]
**Social factors**	
*Connectedness to healthcare providers* - Digital health platform [66] - Telehealth [63] - Telecare [67] - Blood pressure monitor [58] - Computer-Based Self-Management System [85] - Patient portal [79]	*Deterioration of the relationship with healthcare providers* - Telehealth [71] - Telemonitoring [65]
*Connectedness to other people* - Multi-site videoconferencing for home-based education [59] - Telehealth [71] - ICT [80] - Active video games [75]	
*Support from partners and relatives in the use of technology* - Telehealth [71] - Telemonitoring [65]	*Family and social networks* - Robots and sensors [76]
	*Fear of a weakening of social relations with relatives and healthcare providers* - ICT [80] - Telemonitoring [65]
	*Needing others to use the technology* - ICT [80] - Telemonitoring [65] - Active video games [75]
*Patient’s significant others’ and physician’s recommendations for using technology* - Telemonitoring [65]	*Social stigma about ageing and reliance on technology* - ICT [80]

The identified factors were grouped into five domains, which have been used to organise the results: demographic and socioeconomic factors, health-related factors, dispositional factors, technology-related factors and social factors. Evidently, some of the factors placed in one of the five identified domains could simultaneously fall into another domain or present aspects of continuity with other domains. Therefore, the classification made is not rigid, but it is intended to make the results as intelligible as possible.

### Demographic and socioeconomic factors

The impact of age on the adoption of technologies appears to be conflicting, considering demographic and socioeconomic patients’ characteristics. Several studies indicate that older age is a barrier to the utilisation of technology [81, 82, 85], mainly when it comes to using ICT tools for communication with healthcare providers or health education. However, other research did not find that increasing age is a limiting factor for technological interventions, including devices such as a pedometer [73], mobile applications for reporting health outcomes [84] and a telemedicine service [62].

In contrast, the impact of educational level on technology use appears to be more consistently supported by the literature [7, 8, 71, 73, 82]. Well-educated older adults seem to have an advantage in adopting various technologies [73, 82], whereas poor education limits their use and acceptance [71]. An additional factor that has hindered the adoption of technologies by the elderly is related to the economic aspect of technology use. Several investigations consider the cost of technology as a barrier [58, 69, 85], as well as having a low income or not having an adequate socioeconomic status [74]. In the same vein, another study highlights the repercussions of low socioeconomic status, such as the absence of an Internet connection or insufficient space in the household for technological devices [58]. On the other hand, home-based technologies offer the advantage of being cost-effective, as they allow people to save time and money on transportation to medical examinations [59, 65, 74, 79].

### Health-related factors

Studies examining factors associated with the health status of the elderly converge, highlighting that age-related physical limitations can be a significant barrier to the adoption of technological interventions. More specifically, the analysis has revealed a high prevalence of sensory impairments, such as poor vision [71] or hearing loss [76], motor deficits [73] and cognitive disorders, including poor memory [58, 71], limited learning skills [71] and general cognitive degeneration [76].

Furthermore, the lived experience of various health conditions plays a significant role in determining older adults’ acceptance of technology, both positively and negatively. On the one hand, technology seems to be more likely to be accepted if it has been adopted in the early stages of the disease [65]. On the other hand, research has underlined the detrimental effect of comorbidities or complex health conditions on the adoption of technology [60, 66, 86]. Different types of long-term diseases can also affect technology acceptance. For example, Rodríguez-Fernández et al. [86] found that a history of cancer, arthritis and hypertension was positively associated with the effective utilisation of telemedicine. On the contrary, depressive symptoms were negatively associated with it, as well as technical difficulties in using telemedicine were associated with a history of diabetes, heart disease and anxiety symptoms. States of severe anxiety about their illness appear to hinder the use of technology, as observed in the study conducted by Middlemass et al. [65]. Whereas a complex disease experience may lead to perceiving the activity of controlling one’s condition as ‘work’ [73] or an ‘extra burden’ [66], other studies have indicated that self-monitoring technologies appear to be capable of triggering a virtuous circle. In fact, older patients reported greater awareness and understanding of their condition [65, 71], as well as an improvement in health and quality of life through the support they received [66, 69]. Continuous monitoring enables both patients and healthcare providers to gain insights and learn about the medical condition, as well as to make timely adjustments (e.g., modifying diet or medications) [55, 66, 69, 77].

### Dispositional factors

Further aspects are investigated in the literature pertaining to dispositional factors, specifically the beliefs, attitudes and behaviours exhibited by the ageing population towards technologies. A conservative mindset, as well as a strong attachment to daily routines, have been identified as obstacles to the adoption of various technologies [71, 76, 79]. Similarly, a lack of motivation and interest in learning new ways to manage their conditions is an important barrier [60, 64, 65, 71, 76, 79]. Resistance to the adoption of technology may also be the result of a general fear of using new services that could potentially alter their lives [80]. Additionally, specific fears, such as the fear of online fraud [71], the fear of sudden device malfunction [58, 80] and the fear of making mistakes when using healthcare devices, can contribute to this resistance [75].

In addition, privacy seems to be a significant factor in hindering access to technology, especially when it comes to the use of the Internet for health information seeking [71] and patient web portals [78, 83]. Since the ageing population tends to be unfamiliar with new technologies, training is a crucial factor in promoting and enhancing access to eHealth. The knowledge necessary to use technology can be gained through targeted training [58] or acquired through previous experience [74, 80]. However, reluctance to learn how to operate technology, as well as perceived difficulties in the learning process, can make that process challenging.

### Technical factors

As for technology-related factors, the analysed publications consistently emphasise the importance of the perceived ease of use of the various technologies discussed [59, 74, 82, 80, 58–79, 61]. Conversely, the perception of difficulties in using devices or programmes may hinder the acceptance of such technologies [60, 75]. The reviewed studies highlight several technical factors that patients report as problematic. These include connection problems [59, 60, 65], which are particularly evident for patients living in rural or isolated areas. Additionally, interface design issues, such as low-quality graphics [85] and unclear navigation buttons in portals [79, 85], are also mentioned.

Therefore, a simple design [80] and clear presentation and organisation of information are features that facilitate the use of technology [85]. Besides, the technical factors that promote or hinder access to health technologies vary depending on their specificities and their different uses. For instance, in the case of wearable devices, it is important for older people that the technologies are non-invasive and that users perceive them as comfortable [77].

Similarly, in the case of assistive robots, the literature highlights the significance of the robot’s appearance as a relevant factor [76], particularly in terms of how the robot is perceived as trustworthy and likeable [69]. The robot’s speech interaction capabilities have also emerged as particularly important, especially the presence of a speech recognition system and the implementation of robot eye contact and validated gestures to accompany the speech [69].

Interestingly, a technical aspect that seems to encourage the use of both wearable devices and portals concerns the self-tracking functions. These functions include the ability of the tools to set measurable goals for physical activity, quantify users’ health status and activities, receive reminders, allow users to see long-term improvements and share data with healthcare providers [60, 73, 78, 83].

Moreover, another important feature concerns the ability to communicate effectively with healthcare providers, specifically in the case of telehealth. Since telehealth is considered useful in managing medical conditions [58], facilitating factors include the device’s ability to function correctly, transmit accurate and reliable information and provide prompt intervention [58].

### Social factors

Lastly, the analysis has revealed several social factors that are considered relevant based on the literature. First, an important facilitating factor is the perception that health technologies help enhance social ties with nurses and clinicians [63, 66]. Moreover, in the case of telehealth, there is a perception of receiving social support and an improvement in communication between patients and healthcare providers [67].

Research has highlighted ambivalent perspectives on the role played by social networks in the lives of the elderly. On the one hand, having offspring or partners to rely on is considered a facilitating factor for using technology [65, 71], especially when they actively encourage the elderly person to use these devices [65]. In addition, certain technologies seem to enhance connectedness with others; for example, active video games allow people to play with others [75] and videoconferencing platforms are used for home-based education [59]. The latter helped older adults who lived alone to meet new people, and being part of a group allowed them to share information and knowledge with others who had the same condition. Additionally, individuals who suffered from anxiety or depression found it less challenging to participate in online groups rather than to interact with people in person [59].

On the other hand, having more than one person in the house is instead seen as a barrier to technology use because it alters the data registered by sensors [76]. Furthermore, another hindering factor in the use of health technologies is the dependence on others for their use. This is evident in the cases of telehealth [65], ICT [80] or video games for physical activity that require other individuals to participate. The concern that technologies could replace in-person contact/visits by both clinicians and relatives constitutes a barrier, particularly in the case of ICT [80] and telehealth [65].

## Discussion

This study contributes to the understanding of the key factors that influence the acceptance or rejection of health technologies among elderly individuals with chronic diseases or conditions. This is achieved through an analysis of recent empirical literature. Selected studies examine a variety of chronic conditions (i.e. Parkinson’s disease, heart failure, COPD, cognitive impairment, etc.), investigating older adults’ acceptance or rejection of different technologies (i.e. wearable and mobile technologies, telemedicine, assistive robots, etc.). Our review suggests that the technology acceptance or refusal by aged people depends on a wide range of factors, grouped as follows: demographic-socioeconomic, health-related, dispositional, technology-related and social factors.

Regarding demographic and socioeconomic factors, a low educational level [71] and low income [58, 69, 85] have emerged as the main obstacles, which is consistent with previous findings [7, 9]. Instead, the results concerning the impact of age appear more controversial, as some studies included in the review emphasize the negative effect of older age on the adoption of technologies [81, 82], while others did not demonstrate statistically significant differences in technology adoption with advancing age [62, 72, 84]. This inconsistency can be explained by the fact that the samples considered in the examined studies include a target group– individuals over 60 years old– with heterogeneous characteristics. Differences may exist between age groups (for example, between those under 75 and those over 75), as well as within the same age group, given the variation in the aging process and the progression of chronic diseases from person to person.

Therefore, policymakers and technology developers should consider the needs of the most vulnerable and underprivileged social groups, particularly those with low household income and limited educational achievement [22–24]. To enhance access to health technologies, costs should be minimized or even eliminated, for example, by providing support to low-income individuals to access broadband [22, 23]. Specific interventions should be aimed at improving technological competencies and digital health literacy among the elderly. For example, short e-learning courses have been found to be useful for enhancing their technological skills [87] and could be a suitable solution to bridge the digital divide.

Our review highlighted several aspects of older people’s health status, one of the individual-level factors that can negatively impact healthcare technology access. These factors include poor vision [71], hearing loss [76], motor deficits [73], cognitive issues such as poor memory [58, 71] and limited learning skills [71], general cognitive degeneration [76] and the presence of comorbidities or complex health conditions [60, 66, 86]. However, considering the facilitating factors outlined in the review, it is possible to identify some strategies to mitigate these barriers. Firstly, healthcare professionals should recommend the early adoption of health technologies to ensure that older people have the opportunity to learn how to use them before the progression of the disease(s) [65]. Secondly, the developers should provide devices with a design that is as accessible as possible. For example, they could increase screen contrast and use an adequate font size to allow individuals with poor vision to read [63, 65, 79, 83, 84]. Additionally, robots and devices should incorporate sound alerts and a language that can be heard and understood by the elderly with hearing loss and cognitive impairment [58, 59, 69, 76, 85]. Findings also showed several dispositional factors that influence the acceptance or refusal to adopt technologies. For those individuals from older generations who are less familiar with technology, there is a higher likelihood of encountering resistance when it comes to using digital health tools. In fact, a conservative mindset, and a lack of interest in learning new methods of managing their conditions have been identified as significant barriers to the adoption of technologies among the elderly [65, 71, 79]. These findings are consistent with previous studies, which indicate that older adults are often hesitant to embrace new technologies [50, 51, 75]. This reluctance may stem from their familiarity with alternative methods of managing their diseases and their perceived lack of necessity for these devices [88, 89]. Thus, healthcare providers should consider introducing digital health devices to the elderly through personalised and easy-to-understand training [65, 66, 79, 83]. They should also reassure prospective users about privacy issues and provide constant and timely support in case of doubts or device malfunctions [58, 65, 66, 78].

Technical factors emerged as crucial in either promoting or hindering older people’s access to technology. Literature has shown that, in order to be accepted and used over time, devices should be non-invasive and perceived as comfortable by users [77]. They also have functions to measure and quantify body functions and health status, set measurable goals for physical activity, send reminders to users, allow users to track long-term improvements and share data with physicians [75]. As mentioned earlier, technologies should have a simple design [80], and the organisation of the information should be as clear as possible [85]. Technologies for elderly healthcare have to accommodate the needs of individuals with different dis/abilities and physical/cognitive limitations. Furthermore, developers should consider the possibility of involving end users in the design and development process of digital health devices. Because of egocentric bias, younger designers might indeed face challenges in envisioning the product’s usage from the standpoint of an elderly adult [90]. Patients involved in the technology development process are more satisfied and inclined to adopt the technology [58]. In particular, if wearable devices are designed in collaboration with patients, it is easier to avoid issues regarding comfort, size, and ease of fitting, which often pose a barrier to adoption [77]. The involvement of patients in the early stages of technology development can enable the design of more user-centred technologies, and contribute to the early identification of potential issues, thus avoiding the addition of features that patients do not need [31, 58, 66].

The current integrative systematic review has highlighted a domain that is often overlooked but could actually play a crucial role in facilitating technology adoption– the social factors domain. The factors that we have classified in this domain indicate that health technologies can enhance connectivity with others, including nurses, physicians and relatives or patients with a similar health condition. In other words, besides helping to manage the disease, certain technologies can unintentionally have a positive effect by improving the social relationships of older people and encouraging them to embrace technology. Telecare technologies are perceived as useful for receiving social support and improving communication between patients and healthcare providers [67]. Active video games allow people to play with others [75]. Videoconferencing platforms used for home-based education help older adults, especially those who live alone, meet new people and become part of a group [59, 71, 75, 80]. This allows them to share information and knowledge with other people who have the same condition [59]. Additionally, individuals who have experienced anxiety or depression found it less challenging to participate in online groups than to interact with others in person [59]. However, studies have reported that patients fear technologies could replace in-person visits from both clinicians and relatives [65, 80]. Therefore, formal and informal caregivers should receive proper training in the use of healthcare technologies and should be encouraged to alternate between remote and in-person consultations, as this is in the best interest of the older person.

Lastly, our results contribute to a deeper understanding of the ongoing debate surrounding the impact of health technology use, specifically the ‘self-surveillance/empowerment dichotomy’.

On the one hand, the perspective of empowerment positively frames the individual responsibility in managing the disease [45–46]. The ‘empowered patient’ gains power through a better understanding of their illness, which in turn produces a greater sense of responsibility towards self-management of the disease. Patient empowerment thus enhances motivation and adherence to the use of health technologies. As demonstrated in this review, research on platforms for home-based telerehabilitation and health education programs, as well as on wearable activity trackers and active video games, has indicated that motivation can be fostered by various factors.

First, several studies included in the current review have shown that older adults appreciate the self-tracking functions enabled by various types of technology [60, 67, 73, 75, 78]. Health information technologies for self-tracking stimulate individuals to engage in physical or monitoring activities and evaluate their progress towards a goal [60, 73, 75, 78]. Reminders for goal setting have been shown to yield motivational benefits for older adults [60, 66, 74, 75], as well as to receive positive feedback on the accomplishment of personal objectives [69].

Second, m-health devices and telemonitoring platforms also provide patients with the ability to learn more about their disease, and access information and data [60, 73, 75, 78]. Patients perceive greater control over the self-management of their disease and a better understanding of their condition [67]. An increased self-understanding of one’s body and illness can trigger in individuals an attitude of heightened awareness and self-efficacy, which could increase motivation, enable positive coping actions towards self-care, and improve health behaviours. Specifically, seniors show greater motivation to engage with technology when they perceive a clear connection between improved health behaviours and better health outcomes. They can recognize the additional health benefits it offers, such as enhanced autonomy and an improved quality of life [58, 66, 67, 75].

Third, the motivating factors can be socially focused, as an increased sense of connectedness can contribute to generating motivation to adopt technology. Concerning telehealth platforms, the external monitoring of patients by healthcare providers (nurses and physicians) produces a perception of social support and, consequently, can motivate the usage of technology [67]. In the trial conducted by Doyle et al. on a digital health platform [66], the triage service implemented by nurses provided reassurance to participants, as they were monitored ‘behind the scenes’ by healthcare providers who could oversee their parameters and suggest interventions. Additionally, participants expressed appreciation for the social interactions established between them and the nurses [66]. Similarly, digital health platforms, especially those based on telerehabilitation trainings with other participants, can contribute to establishing social interactions with other patients who have the same disease [59, 60, 66, 75]. The participants in the study by Simmich et al. [75] considered the enjoyment derived from playing games with others, specifically through active video games, as a significant motivating factor.

What Petrakaki et al. have referred to as ‘technological self-care’ is the unintended consequence of health technologies that strengthen an individual’s ability to take care of their own health [38]. This includes both personal self-discipline in meeting systemic expectations and the collective encouragement of sharing health knowledge from medical authorities with patients and then disseminating that knowledge to the broader community. This has wider ramifications for the community as a whole [38].

On the other hand, the ‘surveillance effect’, which involves the continuous monitoring of medical data, can remind patients of the negative aspects of their disease. Some may perceive the effort required by these devices as excessive, negatively affecting their motivation to use the devices [66, 73]. Considering the pilot studies, technology appeared to older adults as an added burden to their complicated condition and some participants abandoned the trial due to the onset of health complications [58, 66, 68] or hospitalization [63].

More generally, program completion and adherence are challenging [60, 64, 73], which is particularly evident in long-term trials (one year or more) focused on activity trackers or platforms for rehabilitation training [66, 68]. Adherence seemed high at the beginning of the trial, but over time, there was a decline in compliance for using technology for assisted home exercises [60, 64]. Early withdrawal from the trial can be attributed to various factors, including technical difficulties arising from both structural barriers such as poor connectivity [59, 68] and false alarms generated by the devices [68]. Likewise, frustration stemming from a negative experience with the technology, perceived as too complicated to use, contributes to participant dropout [58, 60, 66].

Consistently, a recent investigation into the reasons for the abandonment of wearable activity trackers has identified six factors [91]. Among these factors, the loss of motivation, which is linked to lower technology acceptance and a negative perception of personal quantification, is one of the most influential [91]. The positive or negative ‘emotional investment’ [92] that people activate when using technologies should be considered for successful adoption.

Some trials examined in the present review employed specific motivational techniques to enhance patients’ adherence to the intervention. These techniques included the use of motivational interviews [60], setting goals and action plans [60, 72], employing reminders and motivational messages to enhance disease self-management and promote self-efficacy [60, 66, 72, 74, 75], as well as providing information on health consequences and assessing outcome goals [60]. In addition, some studies have emphasized the role of caregivers. Their involvement during the trial and the support they provided to the participants ensured better adherence [58, 68]. However, motivational strategies and behaviour change techniques may not be fully effective if they are not accompanied by increased self-reflexivity and self-knowledge of one’s own self and their illness [93].

Moreover, the integration of self-management programs in primary care is often motivated by the need to contain financial pressure and costs associated with managing chronic diseases within healthcare systems [43]. Nevertheless, the implicit transfer of medical responsibilities from healthcare systems to individuals could result in the exclusion of patients who lack access to health technologies or choose not to adopt them [44]. This could potentially worsen inequalities in healthcare access.

Despite the results achieved, this study has some limitations. First, this integrative systematic review excluded certain publications (such as dissertations, conference proceedings, etc.) due to the adopted search strategy. Yet, we believe that the systematic review process adopted, which involved medical, psychological, and sociological databases, as well as three independent researchers who conducted screening and data extraction, ensured a rigorous approach to identifying papers containing consolidated results and relevant information. Second, the analysis focuses on publications written in English, and this may exclude other empirical evidence. Thirdly, an assessment of inter-rater agreement among the authors who reviewed the records has not been conducted. Fourth, this review could not include all studies published before 2012. It is challenging to determine the exact historical moment when certain digital health technologies began to spread, as their implementation depends on various factors (economic, social, cultural, etc.) and can vary in different world regions [94, 95]. Moreover, this review encompasses technologies that have experienced different stages of development. Regardless, to achieve results suitable for current technological developments, it has been decided to establish a timeframe.

Despite these limitations, the selected studies allowed us to conduct an updated analysis of recent literature and identify factors that influence the use of a wide range of technologies by older people with chronic diseases.

## Conclusion

The purpose of the present study was to systematically review the recent literature that addresses the factors related to the adoption or refusal of health technology by elderly individuals with chronic diseases. Moreover, the review provides an overview of the current state of health technologies for elderly individuals with chronic diseases, as well as the specific types of chronic diseases that have been targeted. The findings of the study might help to improve healthcare delivery for this specific population as well as delay disease progression and prevent complications. Besides the positive effects at the individual level, considering the barriers and facilitators that promote the use of health technologies for older individuals leads to a significant decrease in public health expenditure.

Future research aiming to promote technology adoption should therefore consider these factors at different levels: the level of the users, the level of the caregivers and the societal level. In addition, the research will need to delve into the actual effect of the Covid-19 pandemic on the use of technologies. Indeed, on one hand, the pandemic could have acted as a catalyst for the accelerated adoption of technology, enhancing both the implementation and utilization of Internet-based services, such as telemedicine. On the other hand, the pandemic did not address the gap in terms of digital skills, health literacy, and technological competencies among the most vulnerable people, as indicated in a recent systematic review by Elbaz et al. [18].

The study results have practical implications not only for technology developers but also for all the social actors involved in the design and implementation of healthcare technologies, including formal and informal caregivers and policy stakeholders. These actors could use this systematic review to enhance their understanding of the utilisation of technology by the ageing population. This review emphasises the factors that facilitate technology adoption and identifies barriers that impede it, with the ultimate goal of promoting health and independent living.

### Electronic supplementary material

Below is the link to the electronic supplementary material.

Supplementary Material 1: The search strategy used in the electronic databases

Supplementary Material 2: Characteristics of included studies

## Data Availability

The authors declare that all datasets used and/or analysed during the current study are available from the corresponding author on reasonable request.

## Abbreviations

COPD: Chronic Obstructive Pulmonary Disease
ICT: Information and Communication Technology
