# Supplementary material for: Barriers and facilitators to health technology adoption by older adults with chronic diseases: an integrative systematic review
Source: BMC Public Health. 2024 Feb 16;24:506. doi: 10.1186/s12889-024-18036-5 (PMC10873991; doi:10.1186/s12889-024-18036-5)
Supplement: Supplementary file 1 — Supplementary Material 1: The search strategy used in the electronic databases [file 12889_2024_18036_MOESM1_ESM.docx]

Additional File 1: *The search strategy used in the electronic databases*

| Database | PubMed, Scopus, PsycArticles, Web of Science |
| --- | --- |
| Search strategy | #1: elderly OR aged OR older OR older adult* OR frail OR frail older OR frail older OR frail older adult* OR elder OR frail elder* OR geriatric OR elderly people OR old people OR senior OR aging people OR ageing people  #2: technology OR gerontechnology  #3: chronic disease OR chronic illness OR long-term conditions OR chronic conditions  #4: English[language]  #5: ("2012"[Date - Publication]: "2022"[Date - Publication])  #6: #1 AND #2 AND #3 AND #4 AND #5 |
