# Supplementary material for: Barriers and facilitators to health technology adoption by older adults with chronic diseases: an integrative systematic review
Source: BMC Public Health. 2024 Feb 16;24:506. doi: 10.1186/s12889-024-18036-5 (PMC10873991; doi:10.1186/s12889-024-18036-5)
Supplement: Supplementary file 2 — Supplementary Material 2: Characteristics of included studies [file 12889_2024_18036_MOESM2_ESM.docx]

Table S2: *Characteristics of included studies*

| **Authors** | **Objectives** | **Design** | **Country** | **Setting** | **Participants** | **Chronic disease** | **Type of technology** |
| --- | --- | --- | --- | --- | --- | --- | --- |
| AlMahadin *et al.* 2020 [58] | To identify patients and healthcare providers’ preferences, needs, and requirements of wearable devices to monitor symptoms of Parkinson’s disease | Qualitative (focus groups) | U.K. | Home | 12 older adults  Mean age: 73.83 (SD 10.69)  Female: 5 | Parkinson’s disease | Wearable technology |
| Ancker *et al.* 2015 [59] | To investigate how individuals with multiple chronic conditions track their health and medical data through diet and exercise apps or blood  glucose meters, as well as how patients and providers perceive and use data | Qualitative (interviews) | U.S. | Home | 22 older adults  Mean age: mean 64.1 (median 66)  Female: 11 (50%) | Multiple chronic conditions | Health information technologies for self-tracking (diet and exercise apps; blood  glucose meters) |
| Banbury *et al.* 2014 [60] | Evaluation of multi-site videoconferencing as a tool for providing group training to the elderly at home | Qualitative (interviews, focus groups and a journal detailing the technology implementation) | Australia | Home | 52 older adults  Mean age: 73  Female: 28 (54%) | Multiple chronic conditions | Multi-site videoconferencing for home-based education |
| Cajita *et al.* 2018 [61] | To evaluate the perceptions of older adults with heart failure regarding the use of mobile technology for the management of heart failure and identify potential facilitators and barriers to mHealth adoption | Qualitative (interviews) | U.S. | Hospital | 10 older adults  Age range: 66-83  Female: 3 | Heart failure | mHealth |
| Christiansen *et al.* 2021 [62] | To analyse factors affecting mHealth technology use in relation to self-rated QoL among older adults with cognitive impairment | Cross-sectional research | Belgium,  Spain, Sweden | Home | 1.082 older adults  Median age: 75 (IQR = 70–79)  Female: 53.10% | Cognitive impairment | mHealth |
| Doyle *et al.* 2021 [63] | Evaluation of end-user engagement and experiences with a digital health platform, for patient’s self-managing multimorbidity through a 12-month trial | Mixed method (interviews and questionnaires) | Ireland,  Belgium | Home | 120 older adults (Ireland=60; Belgium=60)  Ireland:  Mean age: 74.23 (SD 6.4)  Female: 40%  Belgium:  Mean age: 73.61 (SD 6.49)  Female: 28% | Multiple chronic conditions | Digital health platform |
| Duroseau *et al.* 2017 [64] | To assess patient opinions about technology-based tools, focusing on age-related differences | Cross-sectional research | U.S. | Home | 109 older adults  Mean age: 71.9 years (SD 9.3)  Female: 41.7% | Parkinson’s disease | ICT (email, online tools, video education) |
| Gellis *et al.* 2012 [65] | Examining the impact of a telehealth intervention among older adults diagnosed with heart failure or COPD | Randomized controlled trial | U.S. | Home | 115 older adults  In the intervention (N= 57)  Mean age: 80.1 (7.8)  Female: 62,7%  Control group (N= 58)  Mean age: 78.3 (6.9)  Female: 68,6% | Heart failure and COPD | Telehealth |
| Ha and Park 2020  [66] | To investigate the acceptance of technology among older Korean adults with multiple chronic health conditions and to examine factors associated with technology acceptance | Quantitative (survey) | South Korea | Home | 226 older adults  Mean age: 79.44  Female: 66.37% | Multiple chronic conditions | ICT |
| Herkert *et al.* 2021 [67] | To examine the completion and adherence rates of a home-based exercise program for patients with advanced cardiopulmonary disease | Mixed method | Netherlands | Home | 10 older adults  Median age: 71 (IQR 63-75 years)  Female: 5 | Combined advanced chronic cardiac and pulmonary diseases | Telerehabilitation |
| Jiang *et al.* 2022 [68] | It explored the perceptions and experiences of older patients and healthcare providers in the application of telehealth and online health information to chronic disease management | Qualitative (interviews) | China | Not specified | 29 older adults  Mean age: 70.6 (6.4)  Female: 10 | COPD | Telehealth;  Internet for online health information |
| Jo and Hwang 2021 [69] | To identify the psychological factors affecting the acceptance of ICT services (e.g. Medication Services and Reminders, Object Location Assistance and Reminder, Motivation for  Physical Activity, Fall Detection and Alerting), and the service-related preferences/priorities in South Korean older adults | Qualitative (focus group, interviews) | South Korea | Home | 12 older adults  Aged 65–75 years  Female: 50% | Hypertension, Diabetes, Backache, Angina Pectoris, Arthritis | ICT services |
| Johnson *et al.* 2014 [70] | To describe the KSERA system, how it was developed and validated | Mixed method | Austria, Israel, Italy | Test scenarios | 6 participants  Mean age: 70  Female: 4 | COPD, hypertension, or diabetes | Smart home technology and a socially assistive robot |
| Metting *et al.* 2018 [71] | To investigate patients’ perspectives on Patient Web Portals for disease self-management | Qualitative (focus group) | Netherlands | Home | 29 older adults  Mean age: 65 (SD 10)  Female: 13 (45%) | COPD, Asthma | Patient Web portal |
| Middlemass *et al.* 2017 [72] | To explore patients' perceptions and experiences of using home telemonitoring equipment and comparing the results with the Health Information Technology Acceptance Model | Qualitative (interviews) | UK | Home | 21 older adults  Aged ≥60 years | COPD and heart diseases | Telemonitoring |
| Mitseva *et al.* 2012 [73] | To present the outcomes from intermediate evaluations pertaining users’ satisfaction with the system | Randomized control trial | Denmark, Finland, Grece, UK | Home | 71 older adults  Intervention group (N=45)  Mean age: 77.38 (SD 8.060)  Female: 67.6%  Control group (N=26)  Mean age: 80.00 (SD 8.23)  Female: 61.5%  Informal caregiver (N=71) | Cognitive impairment | Home assistive technology services |
| Nancarrow *et al.* 2016 [74] | Evaluate the effect of a telemonitoring service on older adults with chronic disease | Mixed methods (survey, interviews, focus groups) | Australia | Home | 200 older adults  Mean age 74.8 (SD 8.2)  Female 58,5% | Multiple chronic disease | Telecare |
| Ondiege and Clarke 2017 [75] | To investigate the use of Near Field Communication for identification in a multi-user environment. | Mixed method: (phase 1: pre-trial focus groups); (phase 2: trial); (phase 3: focus groups) | UK | Home | 40 older adults  Aged over 65 | Hypertension | Blood pressure monitor |
| Or and Tao 2012  [76] | To evaluate a new computer-based self-management system interface for older adults with chronic diseases, using a paper prototype approach. | Mixed method (questionnaire, video recording) | Hong Kong | Home | 50 older adults  Mean age: 71.6 | Hypertension, Diabetes, Heart disease, Asthma, Prostatitis, Hypotension | Computer-Based Self-Management System |
| Portz *et al.* 2019 [77] | By adopting the Technology Acceptance Model, the aim is to understand user interface and user experience of a portal for managing chronic conditions | Qualitative (focus groups) | U.S. | Home | 24 older adults  Mean age: 78.41 (SD 5.4)  Female: 17 (71%) | Multiple chronic conditions | Patient portal |
| Price-Haywood *et al.* 2017 [78] | To investigate the relationship between e-health literacy and the use/non-use of a portal technology for self-care, and to identify barriers and facilitators to using the portal | Cross-sectional survey | U.S. | Home | 247 older adults, among which Portal users (N=137)  Portal non-users (N=110)  Mean age:  Portal users: 63.4 (SD 6.7)  Portal non-users: 65.2 (SD 7.5)  Female: 62,5% | Hypertension and diabetes | Portal technology for self-care |
| Reading Turchioe *et al.* 2020 [79] | Evaluating the patient perceived usability of a mobile application for reporting health outcomes, and measuring differences in usability by age | Cross-sectional survey | U.S. | Home | 168 older adults  Age < 65 N=105 (62.5%)  65–74 N=46 (27.4%)  ≥ 75 N=17 (10.1%)  Female:  62 (37%) | Heart failure | Mobile application |
| Robinson *et al.* 2020 [80] | Through the unified theory of acceptance and use of technology,  it has been investigated if age moderates multiple factors that influence the adoption of a physical activity device | Secondary data analysis | U.S. | Home | 59 older Veterans  Mean age: 68.66 (SD 8.93)  Female 2% | COPD | Pedometer |
| Rodrıguez-Fernandez *et al.* 2022 [81] | To investigate association between multiple chronic conditions and “telemedicine readiness” | Cross-sectional study | U.S. | Home | 3379 older adults  Any Readiness Factors (N=1145)  Age <80 776 (67.8%)  ≥80 369 (32,2%)  Female: 595 (52%)  No Readiness Factors (N=2234)  Age <80 1097 (49.1%)  ≥80 1137 (50.9%) Female: 1334 (59.7%) | Multiple Chronic diseases | Telemedicine |
| Simmich *et al.* 2021 [82] | Evaluated attitudes towards wearable physical activity trackers, and barriers and motivators to playing games, for rehabilitation | Qualitative (interviews) | Australia | Home | 19 older adults  Mean age: 70 (SD = 6)  Female 11 (58%) | COPD | Wearable technology;  active video games |
| Smaerup *et al.* 2016 [83] | To evaluate whether elderly patients with vestibular dysfunction are able to preserve physical functional level, quality of life, and reduction in dizziness, when assistive computer technology is used in comparison with printed instructions | Randomized controlled trial | Denmark | Home | 57 older adults  Intervention group Mean age: 79.39 (SD 7.63)  Female: 60.7%  Control group  Mean age: 78.93 (SD 6.58)  Female: 65.5% | Vestibular dysfunction | Computer-Assisted Home Training Program |
| Søraa *et al.* 2021 [84] | To investigate a system called eWare, where a flowerpot robot called “Tessa” works in symbiosis with a sensor technology “SensaraCare.” | Qualitative (interviews) | Norway | Home | 8 older adults  Age range 66-89 years | Cognitive impairment | Robots and sensors |
| Zulfiqar *et al.* 2020 [85] | To evaluate the use of the MyPrediTM remote monitoring platform | Quantitative (questionnaire) | France | Internal medicine unit at the University Hospital of Strasbourg | 36 older adults  Mean age: 81.4  Female: 15 | Cardiovascular disease (31 patients); hypertension (23), lung disease (13); asthma/COPD (11); diabetes (14); solid tumours (4) | Telemedicine |
| Zulfiqar *et al.* 2021 [86] | To evaluate the use of a remote monitoring platform and to experiment with this telemonitoring solution for diabetic elderly patients affected by the SARS-CoV-2 virus to prevent glycaemic disorder risk | Quantitative (questionnaire) | France | Department of Internal Medicine, Diabetes, and Metabolic Disorders at the University Hospital of Strasbourg | 10 older diabetic COVID-19 adults  Mean age: 84.1  Female: 4 | Diabetes | Telemedicine |
